# A Review of Modern Surgical Hair Restoration Techniques

**DOI:** 10.4103/0974-2077.41150

**Published:** 2008-01

**Authors:** Richard C Shiell

**Affiliations:** *14, Jennings Street, Sandringham, VIC, Australia*

**Keywords:** Follicular unit, Hair, Transplantation

## Abstract

The field of hair restoration has had a chequered history. From the days of punch grafting to the present day techniques of follicular unit hair transplantation, the field has seen a number of new advances. This article reviews these developments.

## HISTORICAL BACKGROUND

Hair loss due to disease, scarring and in particular androgenetic alopecia, seems to have troubled members of the human race since the dawn of history. A prescription for restoring hair is included in the 1500 B C. Ebers papyrus of ancient Egypt.

Modern cosmetic hair transplant surgery dates from the work of New York Dermatologist Norman Orentreich in the early 1950s.[1] The technique was in fact much older than this and Japanese dermatologists Sasagawa,[2] Okuda,[3] Tamura[4] and Fujita[5] were using small autografts containing hair follicles for the correction of scars and cicatricial alopecias. It is not known whether they used these techniques for the correction of androgenetic alopecia, but if they did, they certainly did not mention it in their medical papers. In any case their publications, written in Japanese did not reach Western eyes for decades.

Orentreich re-discovered the technique while investigating whether various common skin conditions showed donor or recipient site dominance. Word quickly spread about this wonderful new technique and he soon had many disciples in America and Europe. By 1970, the technique known as “punch grafting” was being performed by small numbers of dedicated practitioners in many countries of the world.

Orentreich's 4.0-mm punch graft method remained the basic procedure until 1975, although some surgeons used smaller grafts at times. The desirability of using smaller punch grafts had certainly occurred to many doctors, but at that time, these small grafts were of unreliable quality when cut with a biopsy punch and never gained the popularity of the larger units. Popularity of punch grafts meant that a generation of patients went around with unnatural “doll look”.

In hindsight, it can be said that it was indeed regrettable that the work of Dr. Hajime Tamura of Japan was not more widely known. Here is an English translation of excerpts from one of his papers written in 1943 and published in the *Japanese Journal of Dermatology and Venereology* at the height of the Pacific War.

*From 1937 onwards I tried the following method on 136 cases including cases of atrichia vulvae, hypotrichosis and cicatricial alopecia and finally confirmed that the grafting of single live hairs is possible*…….

The scalp is excised in a “ship-shaped” manner, leaving the hair 3-4 mm long and sutured immediately. The excised scalp is cleansed in physiological saline and the subcutaneous fat is excised. It is then dissected into small pieces with scissors cutting parallel to the direction of the hair shafts. In the case of single hair grafting the surrounding tissue must be included.

The recipient sites are made with a 1 mm punch or a thick injection needle and the hairs are inserted one by one. Sterilised gauze soaked with olive oil is put over the operated region and a bandage is applied.

The grafted hairs fall out after 2-3 weeks but afterwards the hairs regrow from the same region.

A donor is better if it is as small as possible. The reason for this is that in a big donor graft the hairs grow in a bundle in a most unnatural manner. It is best to conduct the operation with single hairs entirely so that the growth cannot be distinguished from natural growth. Unfortunately the method is very complicated and difficult and therefore I usually use a combination of small grafts into 1 mm punch holes and single hairs into needle sites.

It is indeed remarkable how closely this description resembles the technqiue used today!

## HAIR RESTORATION TECHNIQUES

As the Japanese discoveries were not then known, Western surgeons used several available plastic surgery techniques to re-distribute the donor hair. There are three broad categories of surgical restoration procedures. These may be summarised as follows:

1. scalp flaps (advancement flaps, rotation flaps and free flaps),
2. surgical excision (alopecia reduction), and
3. free autografts of hairy scalp from the well-haired to the bald area.

All three categories of operation are still performed at the time of writing (2008), but the most generally accepted are the autograft techniques known as “*micro-grafting*”, “*mini-grafting*” and, in particular, “*follicular unit transplantation*”.

## SCALP FLAPS

Small pedicle flaps and even free strip grafts of donor scalp had been employed for decades for scar correction on the scalp and eyebrows and had a resurgence after 1975 largely due to the work of J. Juri in Buenos Aries.[6] His long scalp flaps eliminated the curious tufted appearance of a punch graft hairline, but they were still not always popular with patients. This was because of their higher failure rate and even when successful, the frontal hair growth was frequently unnatural in density and direction. In the current practice, the routine use of scalp flaps remains restricted to the hands of a select few individuals such as the Juri brothers in Argentina, Patrick Frechet in France and Mayer and Fleming in the USA.

## ALOPECIA REDUCTION SURGERY

An interesting and logical spin-off from scalp flap surgery was the development of the alopecia reduction operation around 1977.[7] Alopecia reduction procedures could be rapidly learnt and had a high safety factor. A wide number of variations quickly became available and the procedure remained enormously popular for a decade or more. Conflicting camps arose between those who favoured lateral or central reductions.

Morrison, Norwood and Shiell published a paper on *“The Complications of Scalp Reduction”* in 1984,[8] but these warnings went largely unheeded for another decade. The major problems with alopecia reductions were cosmetic. The shape of the residual bald area became increasingly irregular and more difficult to conceal with each additional reduction procedure. In addition, the scalp had a surprising capacity to stretch and much of the initial baldness reduction was lost over subsequent months as the phenomenon titled “stretch-back” consumed up to 50% of the initial gain. Even when all the bald area was excised, one still had the problem of future expansion of the baldness which could expose the old scars.

Frechet introduced his *Triple Flap* procedure in 1989 in an attempt to correct the central slot.[9] He also developed the *Frechet Extender*, a device which was inserted under the skin where it remained for 30 days producing continuous traction on the hair bearing scalp.[10] This not only prevented stretch-back, but also produced additional tissue-creep enabling further tissue to be removed after 30 days. Gerald Seery of the USA advocated the attachment of the advanced scalp to the galea by sutures or a small galeal flap.[11] He claimed that this significantly reduced stretch-back without the introduction of any internal foreign body requiring later removal.

However, these developments in flaps and scalp have lagged behind the advances in graft techniques and the era of alopecia reduction seems to have passed. It remains to be seen whether surgeons of the future, using improved techniques and better case selection will be able to stimulate a new era of alopecia reduction.

## ADVANCES IN AUTOGRAFT TECHNIQUE

Punch grafting remained popular throughout this entire period, but the use of the hand and motorized skin trephine diminished as surgeons switched to square donor grafts cut from long donor strips prepared with multi-blade scalpels. This was not only much quicker, but also eliminated the risk posed by atomized blood particles that spun off the rapidly revolving mechanical punch. This was particularly a worry once the Acquired Immune-Deficiency Syndrome (AIDS) was shown to result from a blood-borne virus.

From the early 1980s, small grafts were produced by dissecting the traditional 4 mm plugs or squares into halves or quarters. These grafts still had up to eight hairs however and still appeared quite tufted when working with coarse black donor hair. Carlos Uebel in Brazil[12] and the Moser Clinic in Vienna[13] advocated large sessions of even smaller grafts containing 3-4 hairs, cut from a donor strip and inserted into slits made with a No 11 blade.

The success and acceptance of this mini/micrografting finally brought the passion for alopecia reduction and 4 mm punch grafting to a halt. At last we had a technique which was safe, relatively easy to learn and produced a result which was popular with patients and surgeons alike. There was a down side as the new technique was much more labour intensive, requiring many hours for the dissection and implantation of 1000 small grafts. The surgeon spent only 1-2 h with the patient and most of the arduous repetitive work was performed by the specially trained surgical assistants.

The labour factor increased again when Dr. William Rassman of Los Angeles pushed session sizes to over 3000 mini-grafts in some cases. This required a team of one surgeon and up to 10 assistants for a total work time of some 80 man-hours.[14] To speed up the production of small grafts, multi-blade knives for the cutting of donor strips acquired up to 10 blades. These could be spaced as close as 1 mm apart but required considerable skill (and strength) to use effectively. Automatic dissection devices were also developed by Boudjema in France in 1992 and Dr. Tony Maugubat in the USA in 1996.[15]

## MICROSCOPE-AIDED DISSECTION

Many surgeons were alarmed at the degree of follicular transection that was occurring with the “blind” cutting of multiple strips with multi-blade knives and the new dissection devices. It was estimated that up to 25% of follicles were traumatized in some cases and that, even with the most skilled surgeons, this figure was around 10%.[16] In their defence, the multi-strip surgeons quoted the work of J.C. Kim of Korea who demonstrated experimentally that most transected follicles eventually regrew hair.[17]

Strip dissection under stereoscopic microscopes had been introduced by Dr. Bob Limmer of Texas in 1987and gave the operator an unprecedented view of the excised scalp tissue and the individual hair follicles.[18] Microscopic dissection averaged only about 150-200 grafts per hour however and greatly increased the number of staff members required for each procedure. As a result, there was much initial resistance to the new microscopic methods and professionals were slow to take up this meticulous technique. Later, however, David Seager of Toronto[19] wrote extensively and eloquently about the technique and it was taken up further by doctors Bernstein and Rassman[20] and many others. Dissection teams of 10 or more assistants became common and an additional 2-3 assistants were required for graft implantation.

There was a downside to this development too. It was no longer possible for a cosmetic surgeon with a casual interest in hair restoration to perform these new procedures at a high standard. Unless he had a regular flow of hair patients, it was not feasible for the surgeon to assemble, train and keep a large team of surgical assistants together and therefore technique became restricted to few dedicated teams.

## ALTERNATIVE APPROACHES

In Korea, a alternative approach for speeding up the process of transplantation was developed by Choi *et al*.[21] The follicular units or bundles still had to be carefully prepared by hand, but they were implanted with the aid of a mechanical implantation device. The Choi Implanter is the most ingenious device into which follicular bundle containing 1-4 hairs may be loaded. The needle is inserted into the scalp and the plunger pressed to implant the graft. With a three-person team of two loaders and one planter about 12 grafts per minute or around 700/h can be implanted. As an alternative regime, the fine slits can be pre-made by the surgeon and the assistants “fill the holes” with the aid of the implanter at some later time. This enables very close spacing of the grafts and the surgeon remains in full control of the spacing and direction of each implant. Professor Jung Chul Kim, also of South Korea, has developed his own version of the Choi implanter that has a different method of action and disposable needles. Surgeons outside of Asia are slowly showing interest in both these devices and they are now being used in Greece and other centres.

A Hair Implanter Pen was developed by Dr. Pascal Boudjema of France[22] and mechanical implantation devices have been developed by Dr. Bill Rassman[23] and Dr. Barry Markman of the USA. These do not appear to have gained many adherents at the time of writing.

## DONOR SCARS

The switch from individual 4-mm donor plugs sites to strip excision of the donor site in the 1980s lead to complaints from patients (and their hairdressers) about residual linear scarring at the back and sides of the patient's scalp. Wound tension was a major factor in causing wide scar. However, wide scar often occurred even when there had been minimal tension during closure and it is possible therefore, that there are significant individual variations in healing characteristics of human skin, probably of a genetic basis.

To overcome this problem of donor scar, there have been two developments:

1. A return to the removal of individual grafts, this time of 1.0 mm (or even less) follicular-unit dimensions. This is technically difficult and may result in damage to the follicle during extraction of the follicular unit. This has become known as follicular unit extraction or FUE.[24]
2. A refined donor closure technique was developed as a spin-off from the frontal flap techniques of two decades earlier. This, known as the *“trichophytic”* closure, was designed to allow hairs to grow through the residual scar.[25] This was achieved by snipping a 1-mm ledge of epidermis off one edge of the donor site before the closure of the wound, in the expectation that the underlying hair will grow through the linear scar. This is usually very successful providing a resultant scar, which is no more than 2 mm wide.

## LESSONS THAT HAVE BEEN LEARNT SO FAR

First, it would be a mistake to think that early methods were universally primitive and the results coarse and unaesthetic in all cases. While there were many poor results from surgeons in the learning stage, some of the results from older 4-mm plug, flap and reduction procedures were indeed satisfactory. Patients with dense fair or grey hair generally obtained excellent results, especially after careful hair styling. The problem was always with those who had dark hair and a pale scalp. It was in these individuals where the tufty nature of these early efforts was most apparent. This was almost the sole reason for which the scalp hair flaps and later the micro-grafts were developed.

Second, there is a long-learning curve with most cosmetic surgery procedures. Even those techniques which seem simple at first glance, often take up to 2 years of practice before a dermatologic surgeon can achieve consistent satisfactory results. This is true of hair restoration surgery also and one needs constant practice to achieve the skill level needed to cut and plant high quality grafts with ease and reliability.

Small grafts have provided the opportunity to create almost undetectable new hair coverage. However, this has also meant that the hair transplantation has become labour intensive and also expensive. Interestingly, hair-loss patients have become a lot more “fussy” and demanding in their requirements. Many experienced surgeons have noticed that the patient dissatisfaction rate has actually increased in the past decade, even though the results have been greatly improving.[26] This is particularly true when some surgeons yield to the temptation to promote themselves in newspapers or on the Internet as “gurus” promising results verging on the miraculous, and raising the expectations of the patients to unreasonable levels.

## SUMMARY

It is important to understand that, in all hair restoration procedures, skill of the surgeon is as important as the precise surgical method. An experienced and skilled surgeon can usually anticipate cosmetic and psychological problems before they arise and take steps to avoid these. Hair transplantation still remains as much an art form as a science and those who forget this fact in a rush to technology and increased staff numbers, are doomed to disappointment.

The grafting of follicular units certainly reigns supreme at present, with meticulous dissection under stereoscopic microscopes, as the “gold standard”. It is difficult to see this being superseded in the future. It is essential that the surgeon remain attuned to the individual sensitivities and requirements of each patient however and not regard him as a mere “client” to be processed through a semi-automated surgical production line.

A limitless supply of cloned hair follicles is a distinct possibility within the next few years. The use of the drug finasteride has already made a big transformation in our approach to those in early hair loss and it is most likely that chemical and genetic correction of baldness will ultimately replace the need for surgical hair transplantation.

## TRAINING

Medical graduates wishing to learn more about methods of hair replacement are now in the most fortunate position. There are excellent textbooks available[27,28] and an annual 4-day meeting organized by the International Society for Hair Restoration Surgery. In addition, there are at least two hands-on workshops held each year in various parts of the world and the International Society has an excellent bi-monthly journal, *Hair Transplant Forum International* in which the latest ideas and techniques are constantly under discussion.
